# Neighborhood socioeconomic measures and disparities in hospitalization for atrial fibrillation among medicare beneficiaries: a national county-level analysis

**DOI:** 10.3389/fcvm.2026.1779097

**Published:** 2026-04-10

**Authors:** Reuben Odai, Chukwuemeka Aghasili, Smith Frimpong, Laiba Sajjad, Anthony Alchaer, Wan-Chi Chan, Ibrahim Touffaha, Andy Le, Freidy Eid

**Affiliations:** 1 School of Medicine Wichita, University of Kansas Medical Center, Wichita, KS, United States; 2 Department of Internal Medicine, Geisinger Wyoming Valley Medical Center, Wilkes-Barre, PA, United States

**Keywords:** atrial fibrillation, healthcare disparities (MeSH), hospitalization, medicare, social determinants of health (SDOH)

## Abstract

**Objectives:**

To examine associations between Social Vulnerability Index (SVI), Social Deprivation Index (SDI), and median household income, and rates of hospitalizations for atrial fibrillation (AF) among Medicare beneficiaries.

**Methods:**

We analyzed hospitalizations with AF as the principal diagnosis among Medicare beneficiaries (aged ≥65) across 3,116 US counties (2019–2021)**.** Age-standardized hospitalization rates were obtained from the CDC's Atlas of Heart Disease and Stroke. We examined associations between social determinants and hospitalization rates overall and stratified by sex and race/ethnicity using correlation and linear regression analyses.

**Results:**

The overall rate of hospitalization among patients with AF was 575 per 100,000 beneficiaries. Each 0.01-point increase in SVI and 1-point increase in SDI was associated with 1.6 and 1.7 additional hospitalizations per 100,000, respectively (*p* < 0.001). Each $1,000 increase in median household income was associated with 1.8 fewer hospitalizations (*p* < 0.001). Income effects were strongest among women (−2.3 per $1,000) and persisted across all racial/ethnic groups. SDI demonstrated superior model fit (AIC: 12,967) compared to SVI (AIC: 13,000) and income (AIC: 13,069), explaining 6.2% of variance in hospitalization rates.

**Conclusions:**

Neighborhood social determinants are strongly associated with AF hospitalization rates among Medicare beneficiaries despite near-universal coverage, with pronounced sex disparities. These findings support the use of county-level socioeconomic indices for geographic surveillance, while underscoring that patient-level longitudinal studies are needed to identify the specific drivers of these associations before clinical or policy interventions can be recommended.

## Introduction

While traditional cardiovascular risk factors are well-established predictors of atrial fibrillation (AF), emerging evidence suggests that social determinants of health, including neighborhood socioeconomic conditions, are associated with AF outcomes (1). Socioeconomic disadvantage may be associated with AF-related health outcomes through reduced access to preventive care, delayed diagnosis, and barriers to anticoagulation therapy (1, 2).

Despite Medicare providing near-universal coverage for adults aged ≥65 years, substantial geographic and demographic disparities in AF hospitalization rates persist. In this ecological study, we examined associations between three validated measures of neighborhood social determinants: Social Vulnerability Index (SVI), Social Deprivation Index (SDI), and median household income, with AF hospitalization rates among Medicare beneficiaries across U.S. counties to characterize place-based disparities and generate hypotheses for future investigation.

## Methods

We conducted a retrospective cross-sectional analysis of hospitalizations for AF among Medicare beneficiaries aged ≥65 years across U.S. counties from 2019 to 2021. Age-standardized hospitalization rates per 100,000 beneficiaries, stratified by sex and race/ethnicity, were obtained from the CDC's Atlas of Heart Disease and Stroke (3). Hospitalization with atrial fibrillation was defined according to the CDC Atlas of Heart Disease and Stroke using Medicare Provider Analysis and Review (MEDPAR) Part A data as an inpatient hospitalization with atrial fibrillation listed as the principal (first-listed) diagnosis (ICD-10-CM I48.0–I48.2, I48.91). Social determinants were measured using SVI 2018 (4), SDI 2019 (5), and median household income. Institutional review board approval was not required as all data were publicly available and deidentified.

The SVI is a composite measure (range 0–1) from the American Community Survey (ACS), incorporating 16 U.S. Census variables across four domains: socioeconomic status, household composition and disability, minority status and language, and housing type and transportation. The SDI (range 0–100), also derived from ACS, comprises seven characteristics: percent below the poverty line, percent with less than 12 years of education, percent in single-parent households, percent in rented housing, percent in overcrowded housing, percent without vehicle access, and percent of non-employed adults aged 16–64 years. Higher values indicate greater vulnerability/deprivation for both indices. Median household income was obtained from the 2021 U.S. Census Bureau (6), which provided a direct measure of economic resources and purchasing power at the county level.

We performed Pearson correlation analyses and unadjusted linear regression models to examine associations between social determinants and AF hospitalization rates, overall and stratified by sex and race/ethnicity. We did not perform adjustment/sensitivity analyses because harmonized county-level comorbidity and resource measures were not available across the same years for all counties. Therefore, we present unadjusted associations and interpret them as descriptive, with residual confounding likely. To compare the relative strength of each social determinant measure, we calculated Akaike Information Criterion (AIC), Bayesian Information Criterion (BIC), and R^2^ values for each unadjusted model. Lower AIC/BIC values and higher R^2^ indicate better model fit. Analyses were conducted using Stata/BE 19.0.

## Results

We analyzed 3,116 U.S. counties in the United States. The overall rate of hospitalization for AF was 575.0 per 100,000 Medicare beneficiaries (95% CI: 568–582), higher in women than men (597.5 vs. 552.6 per 100,000) and among White vs. Black and Hispanic beneficiaries (599.0 vs. 383.9 and 311.8 per 100,000, respectively).

Correlation analysis revealed significant positive associations between rates of hospitalization and both SVI (r = 0.23, *p* < 0.001) and SDI (r = 0.25, *p* < 0.001), while median household income showed an inverse association (r = −0.18, *p* < 0.001). Each 0.01-point increase in SVI was associated with 1.6 additional AF hospitalizations per 100,000 beneficiaries (*p* < 0.001), while each 1-point increase in SDI was associated with 1.7 additional hospitalizations (*p* < 0.001). Each $1,000 increase in median household income was associated with 1.8 fewer hospitalizations (*p* < 0.001).

Comparing model performance across the three social determinant measures, SDI demonstrated superior fit (AIC: 12,966.6, BIC: 12,978.7, R^2^: 0.062) compared to SVI (AIC: 13,000.1, BIC: 13,012.2, R^2^: 0.052) and median household income (AIC: 13,069.0, BIC: 13,081.1, R^2^: 0.031). SDI explained the most variance in AF hospitalization rates and showed the best model fit by information criteria. The substantial intercorrelation among these measures (SVI-SDI: r = 0.84; SDI-income: r = −0.55; SVI-income: r = −0.47) indicates they capture overlapping but distinct aspects of neighborhood disadvantage. Subgroup analyses revealed differential model performance by race/ethnicity. While SDI demonstrated superior fit for White beneficiaries (AIC: 13,257) and both men (AIC: 12,438) and women (AIC: 13,155), median household income showed better fit for Black (AIC: 3,560) and Hispanic beneficiaries (AIC: 746.6), supporting our finding that income effects were most consistent across all racial/ethnic groups. Women experienced stronger associations with all three measures, particularly with income (2.3 fewer hospitalizations per $1,000 increase) compared to men (1.5 fewer per $1,000). Details of overall and subgroup analyses are presented in Table 1.

**Table 1:** Rates of hospitalization with AF and unadjusted associations with neighborhood indices and income.

Group	AF Hospitalization (per 100,000 beneficiaries), (95% CI)	Rate per 0.01 unit increase in SVI (95% CI)	*p*-value (SVI)	Rate per 1-unit increase in SDI (95% CI)	*p*-value (SDI)	Rate per $1,000 increase in median income (95% CI)	*p*-value (income)
Overall	575 (568–582)	1.6 (1.3–1.8)	<0.001	1.7 (1.5–2.0)	<0.001	–1.8 (–2.2 to –1.5)	<0.001
Men	553 (546–559)	1.1 (0.9–1.3)	<0.001	1.2 (1.0–1.4)	<0.001	–1.5 (–1.8 to –1.1)	<0.001
Women	597 (590–605)	1.8 (1.6–2.1)	<0.001	2.0 (1.8–2.3)	<0.001	–2.3 (–2.7 to –1.9)	<0.001
White	599 (592–607)	2.1 (1.8–2.3)	<0.001	2.2 (2.0–2.5)	<0.001	–1.9 (–2.3 to –1.6)	<0.001
Black	384 (377–391)	0.4 (0.1–0.6)	0.004	0.3 (0.1–0.6)	0.013	–1.0 (–1.3 to –0.7)	<0.001
Hispanic	312 (296–327)	–0.2 (–0.8–0.3)	0.443	–0.3 (–0.7–0.2)	0.312	–0.6 (–1.1 to –0.1)	0.014

AF, atrial fibrillation; SVI: social vulnerability index; SDI:, social deprivation index; CI, confidence interval.

## Discussion

Our study found significant associations between neighborhood social determinants and rates of hospitalization with atrial fibrillation among Medicare beneficiaries, with important sex and racial/ethnic disparities. Higher social vulnerability and deprivation were associated with higher hospitalization rates, whereas higher median household income was inversely associated with hospitalization with AF.

Our model comparison analysis revealed that SDI outperformed both SVI and median household income across all fit metrics, explaining 6.2% of variance in AF hospitalization rates compared to 5.2% for SVI and 3.1% for income. This superior performance may reflect SDI's comprehensive capture of seven distinct socioeconomic characteristics that collectively are associated with healthcare access and utilization. The inverse association between median household income and AF hospitalizations is particularly noteworthy, with each $1,000 increase associated with 1.8 fewer hospitalizations per 100,000 beneficiaries. This relationship was strongest among women and persisted across all racial/ethnic groups, including populations where SVI and SDI showed attenuated associations. Prior research established that social determinants are associated with AF management through limited healthcare access and reduced anticoagulation adherence (1, 2). Our findings suggest these associations are also present within the Medicare population, indicating that insurance coverage alone may not fully eliminate socioeconomic disparities in AF-related hospitalizations.

Women experienced substantially stronger associations between SVI/SDI and hospitalization rates (1.8 and 2.0 per unit increase) compared with men (1.1 and 1.2 per unit increase), with pronounced income gradients (−2.3 vs. −1.5 per $1,000). Women with AF typically present with greater symptom burden and comorbidities, potentially leading to increased hospitalizations in disadvantaged areas where healthcare access may be compromised (7, 8).

The racial disparities present a complex pattern that warrants careful interpretation. While White beneficiaries had substantially higher AF hospitalization rates, all racial/ethnic groups showed inverse associations with income. This may reflect the “AF paradox” of lower diagnosed prevalence in minority populations and the universal importance of economic resources (1). The attenuated or null associations between SVI/SDI and hospitalizations among Hispanic beneficiaries should be interpreted cautiously, as multiple explanations are plausible: underdiagnosis, coding variation, or genuine differences. These explanations remain speculative given our ecological design and hospitalization-based ascertainment.

The strong intercorrelation among social determinant measures reflects overlapping aspects of neighborhood disadvantage. However, median household income's robust and consistent associations across demographic groups highlight economic resources as a fundamental determinant of AF hospitalization risk.

## Public health implications

Our study supports the use of county-level socioeconomic indices for geographic surveillance and health system resource allocation, identifying communities where AF-related hospitalization burden is disproportionately high. By comparing three validated measures: SVI, SDI, and median household income, we demonstrate that all three indices capture meaningfully distinct aspects of neighborhood disadvantage. Importantly, these associations are hypothesis-generating rather than prescriptive: our ecological design cannot distinguish whether the observed patterns reflect differences in comorbidity burden, lifestyle-related risk factors, barriers to outpatient care, or differential rates of procedural admissions. Patient-level longitudinal studies are needed to identify which mechanisms are most actionable before specific clinical or policy interventions can be recommended.

## Limitations

As an ecological study, county-level associations cannot establish individual-level causation. We did not adjust for county-level comorbidity burden, lifestyle factors, or healthcare infrastructure, all of which may confound the observed associations and limit causal inference. Residual confounding could inflate or attenuate observed associations. However, identifying place-based disparities is essential for targeting public health interventions and health system resource allocation. SVI and SDI were derived from different ACS 5-year estimate periods (2016–2020 for SVI and 2015–2019 for SDI), and median income from 2021, introducing a minor temporal mismatch; however, these indices are relatively stable over short timeframes. Reliance on hospitalization data may underestimate AF burden in populations with reduced healthcare access. Race/ethnicity–stratified analyses may be affected by differential county availability/suppression, limiting precision and generalizability. Geographic variation in coding practices may introduce measurement bias.

## Conclusion

This nationwide analysis reveals significant associations between neighborhood social determinants and AF hospitalization rates among Medicare beneficiaries, with median household income showing inversely associated effects across all demographic groups. Important disparities exist by sex and race/ethnicity. These findings suggest that insurance coverage alone may not eliminate socioeconomic differences in AF-related inpatient utilization, but the mechanisms underlying these associations require further evaluation in patient-level and longitudinal studies.

## Data Availability

The original contributions presented in the study are included in the article/supplementary material, further inquiries can be directed to the corresponding author.
